# Don't rock the boat! Do men prefer women leaders who support the status quo?

**DOI:** 10.1111/bjso.70053

**Published:** 2026-02-06

**Authors:** Belle Derks, Francesca Manzi, Colette van Laar, Naomi Ellemers

**Affiliations:** ^1^ Social, Health and Organizational Psychology Utrecht University Utrecht the Netherlands; ^2^ Department of Management London School of Economics and Political Science London UK; ^3^ KU Leuven, Center for Social and Cultural Psychology Leuven Belgium

**Keywords:** gender inequality, gender stereotypes, masculine defaults, queen bee phenomenon, system justification, women leaders

## Abstract

Women remain underrepresented in leadership, particularly in traditionally masculine work settings. At the same time, the visibility of this imbalance has led to growing calls for diversifying leadership. This research examines how both men and women contribute to the preservation or disruption of gender inequality in masculine organizational contexts. Men remain the gatekeepers of change—deciding who rises to the top and under what conditions—while women face the strategic dilemma of fitting in by downplaying inequality (supporting the status quo, sometimes called ‘queen bee behaviour’) or ‘rocking the boat’ by advocating social change (challenging the status quo). Across five experimental studies (total *N* = 887), we examined how evaluators assessed male and female leadership candidates who either supported or challenged the status quo. Results revealed that although men favoured female over male candidates, they consistently preferred women who reinforced the status quo over those who advocated equality. By contrast, male candidates who supported the status quo were penalized, and female evaluators showed no such preferences. These findings highlight subtle mechanisms through which gendered power dynamics are maintained, underscoring both the strategic trade‐offs women must navigate to advance and the conditional nature of men's support for gender equality.

Despite progress, women remain underrepresented in leadership—especially in traditionally masculine settings (Catalyst, 2024a, 2024b). In response, governments, advocates and organizations have launched initiatives ranging from individual‐focused programmes that build confidence, skills and aspirations to structural reforms such as quotas and inclusive leadership policies (Benschop et al., 2015; Kelan & Wratil, 2018; Ryan & Morgenroth, 2024). Pressure to diversify leadership is particularly acute in masculine workplaces and is—implicitly or explicitly—directed at the men who still dominate these environments and their boards (Cortis et al., 2021). Understanding how men respond to calls for gender equality is therefore crucial: in these contexts, men remain key gatekeepers and their preferences and decisions largely determine who advances—and under what conditions.

As men weigh whether and how to support diversity, aspiring women leaders in masculine settings face a different dilemma. They are encouraged to “lean in” and assimilate to masculine norms—signalling ambition, competitiveness and individual drive to prove they can succeed on masculine terms (Kim et al., 2018; Ryan & Morgenroth, 2024)— On the other hand, the very push for greater gender diversity often brings the expectation that women should act as change agents: championing other women, challenging discriminatory practices and modelling an alternative leadership style (Mavin, 2008; Webber & Giuffre, 2019). These competing demands pit individual advancement against systemic change and shape how decision‐makers evaluate women—affecting both women's progression and the success of gender‐equality initiatives.

In this paper, we examine how men in masculine work settings respond to calls for greater gender equality by evaluating female and male leadership candidates who either support or challenge the status quo. While heightened attention to inequality and pressure to diversify may boost men's evaluations of female (vs. male) candidates, we argue they will still prefer women who support the status quo (e.g., adopt traditionally masculine traits, downplay inequality) over those who challenge it (e.g., display traditionally feminine traits, highlight systemic barriers). This research aims to shed light on the dual pressures shaping gender‐equality efforts: the role of majority‐group members (i.e., men) in enacting or resisting organizational change and the strategic trade‐offs that minority‐group members (i.e., women) must navigate to succeed within unequal systems.

## The persistence of gender inequality in leadership

Women now represent about half of the paid labour force in most industrialized nations, and their participation in traditionally masculine roles has risen sharply (Catalyst, 2024a, 2024b; Cheryan et al., 2017; Ortiz‐Ospina et al., 2018). They have also gained leadership ground—holding roughly one‐third of parliamentary seats and board positions in Europe and North America (Catalyst, 2024a, 2024b; UN Women, 2024). Yet women remain underrepresented across corporate, political and academic leadership pipelines, especially at the top (AAUW, 2022; Catalyst, 2024a, 2024b; Krivkovich et al., 2024; UN Women, 2024). Research points less to differences in qualifications or preferences than to persistent gender bias: even when equally qualified, women are less likely to access leadership roles, earn less for equivalent work and face higher rates of mistreatment and harassment (Catalyst, 2018; Cortina, 2008; Joshi et al., 2022; Penner et al., 2023).

These differential outcomes for women and men have been traced back to two key factors: gender stereotypes and the “masculine defaults” embedded in leadership characterizations. Gender stereotypes are culturally shared beliefs that depict women as communal (e.g., kind, sensitive) and men as agentic (e.g., assertive, rational; Ellemers, 2018; Heilman et al., 2024). In traditionally masculine roles—such as leadership—success is largely associated with agentic traits such as dominance, competition and self‐confidence (Cheryan & Markus, 2020). As a result, men are viewed as the natural fit while women are seen as lacking the attributes required to be competent leaders (Eagly & Karau, 2002; Heilman et al., 2024). This perceived mismatch between communal depictions of women and agentic role expectations contributes to discrimination: women must often provide more evidence of their competence to receive the same recognition as men (Biernat & Kobrynowicz, 1997; Koenig & Eagly, 2014; Leslie et al., 2015).

Yet even when women demonstrate competence and fit, they continue to face bias. Women who defy communal gender stereotypes by exhibiting assertiveness, dominance and competitiveness—the very traits valued in leadership roles—are often judged as interpersonally hostile and less likable (Heilman et al., 2024; Rudman & Glick, 2001). This creates a double bind: behaving communally reinforces perceptions of poor fit and low leadership potential, while behaving agentically elicits social penalties for norm violation. Unlike their male counterparts, women in masculine work settings must navigate the tension between their professional and gender identities—and often must work harder to be seen as both competent and adequate.

## Men in masculine work settings: Allies or gatekeepers of the status quo?

As workplace inequality gains visibility and is increasingly scrutinized, calls for change have intensified and initiatives advocating for the advancement of women have become increasingly common—particularly in leadership (e.g., Kratz, 2024; Leslie et al., 2024). Governments, schools and firms have promoted diversity through mentoring, empowerment workshops and structural reforms such as voluntary or mandatory gender quotas (European Institute for Gender Equality, 2022; Krivkovich et al., 2024; UN Women, 2025). These efforts focus particularly on masculine work settings, where disparities and masculine norms are most entrenched (Cheryan & Markus, 2020). In these environments, the responsibility for implementing change often rests with the men who dominate leadership positions. As such, men effectively hold the power to either disrupt or reinforce the status quo through their support for—or resistance to—gender‐equality initiatives (Cortis et al., 2021).

As members of the majority group, men in masculine work settings are uniquely positioned to legitimize and advance gender‐equality efforts (Sherf et al., 2017). When men speak out against sexism or advocate for women's advancement, their actions are often perceived as more credible and objective than when women do the same (Drury & Kaiser, 2014). Male allyship can also foster greater inclusion for women and signal institutional support for equity goals (Moser & Branscombe, 2022). Thus, men have the potential to shift workplace norms and serve as powerful agents of change through their attitudes, decisions and behaviour.

Despite this potential, many men in masculine organizations respond to diversity initiatives with ambivalence or resistance (Kanitz et al., 2024). As beneficiaries of existing hierarchies, they are motivated to justify the status quo (Kay et al., 2009). Pro‐diversity messages and calls for gender equality can trigger status threat—concerns that advancing women endangers men's standing (Dover et al., 2016; Ellemers et al., 2002)—often fuelled by zero‐sum beliefs that women's gains mean men's losses (Iyer, 2022; Zehnter et al., 2021). Such initiatives may also evoke symbolic threats to entrenched cultural values and ingroup morality threats that implicate men in inequality (Stephan & Stephan, 2000).

Organizational narratives can also reinforce men's resistance to change. Many masculine work settings endorse and promote a meritocratic ideology that assumes success is primarily based on individual talent and effort (Cheryan & Markus, 2020). While this narrative appears fair, it often obscures the structural barriers women face—allowing decision‐makers to attribute women's underrepresentation to personal deficits rather than systemic disadvantage (Castilla & Benard, 2010). At the same time, meritocratic beliefs allow men in masculine work settings to maintain a positive self‐image while legitimizing the status quo (McCoy & Major, 2007; Shuman et al., 2023). Even when men endorse diversity, their support may remain largely symbolic or performative (Kutlaca & Radke, 2023; Pietri et al., 2024). For example, organizations may increase female representation in visible roles without making deeper structural changes (Chang et al., 2019; Krivkovich et al., 2024). These surface‐level commitments can be counterproductive as they create the appearance of progress while leaving masculine norms and hierarchies intact.

In sum, while men have the potential to act as influential allies in advancing gender equality, their responses to diversity efforts and calls for greater equality—ranging from active support to passive compliance or resistance—play a critical role in shaping whether change is merely performative or truly transformative.

## Women in masculine work settings: Fitting in or rocking the boat?

While men in masculine organizations must choose between allyship and gatekeeping, women face a different dilemma: whether to assimilate into the existing culture to advance individually or to challenge the status quo and support systemic change. Research suggests that women cope with negative gender stereotypes and masculine defaults through two different strategies. One focuses on collective action and involves relying on their gender group, advocating for cultural change and equal opportunities and questioning masculine norms of success (Iyer & Ryan, 2009). The other strategy focuses on individual mobility and involves distancing themselves from their gender group, supporting meritocratic narratives and aligning with masculine norms of success (Derks et al., 2016; Ellemers et al., 1997; Veldman et al., 2020). These strategies are not mutually exclusive—women may switch between them across contexts or career stages. However, their use is often shaped by gender identification: those who identify more strongly with their gender are more likely to engage in collective strategies, while those who identify less strongly are more likely to prioritize individual advancement (Derks, Ellemers, et al., 2011; Derks, Van Laar, et al., 2011; Iyer & Ryan, 2009). Importantly, although each strategy has its own benefits, they also come with their own set of consequences.

### Rocking the boat: Challenging the status quo through collective action

Women in masculine work settings can challenge the status quo both implicitly, by modelling alternative leadership styles, and explicitly, by directly confronting inequality. Implicit challenges often take the form of communal leadership, which contrasts with traditional models that place agency as the key determinant of success. For example, compared with men, women leaders are more likely to combine agency (e.g., risk‐taking, goal‐setting) with communality (e.g., empathy, teamwork; Eagly & Carli, 2003; Vial & Cowgill, 2022). They are also more likely to foster psychologically safe, collaborative and family‐supportive work environments (Post, 2015; Sargent et al., 2022; Wallen, 2002). These leadership styles not only disrupt the status quo but also support the retention and advancement of junior women (Sealy & Singh, 2010). Importantly, women are expected to bring these alternative styles and perspectives—to “add value” by diversifying leadership—whereas men can rely on conformity to established, agentic norms (Eagly & Karau, 2002).

On a more explicit level, women leaders may challenge the status quo by acting as mentors for other women, advocating for diversity initiatives and speaking out against gender discrimination (Becker et al., 2014; Cortis et al., 2021; Derks, Ellemers, et al., 2011; Derks, Van Laar, et al., 2011; Fritz & van Knippenberg, 2017; Iyer & Ryan, 2009). These behaviours position women as change agents in masculine work settings, with broad downstream consequences: Organizations with a higher representation of women in leadership are less likely to be gender segregated at lower levels (Stainback & Kwon, 2012), have a narrower gender wage gap (Zimmermann, 2022), and are more likely to hire women in lower management positions (Arvate et al., 2018).

Challenging the status quo can carry costs for women's leadership prospects. Women who emphasize communality are often perceived as lacking the agentic qualities stereotypically associated with effective leaders—falling into the well‐documented “think manager, think male” bias (Cheryan & Markus, 2020; Eagly & Karau, 2002; Heilman & Manzi, 2022). In addition, women who explicitly question the legitimacy of gendered workplace norms may be seen as confrontational or disruptive, undermining their chances of success, as people are motivated to defend existing arrangements and derogate challengers (Heine et al., 2006; Jost & Banaji, 1994; Rudman & Glick, 2001). Women face steeper penalties than men for confronting sexism—more likely to be labelled complainers and evaluated negatively (Dodd et al., 2001; Eliezer & Major, 2012). Relative to men and majority‐group members, women and ethnic minorities who advocate for diversity are seen as self‐interested and rated lower in competence and performance (Gardner & Ryan, 2020; Hekman et al., 2017).

Moreover, women who express concern about gender inequality risk being associated with feminism stigma. Feminist‐identifying women are often stereotyped as angry, anti‐male or difficult, and are less likely to be seen as competent or persuasive (Radke et al., 2016). Expressing anger violates prescriptive gender norms that require women to be agreeable and emotionally restrained (Brescoll & Uhlmann, 2008), leading to perceptions of unreasonableness and reducing their likelihood of being seen as fit for leadership (Rudman & Glick, 1999).

In sum, collective strategies can benefit women as a group yet backfire individually by triggering stereotype‐based backlash that diminishes perceived leadership potential. These costs are magnified in masculine organizations where men control most decisions: as gatekeepers, male evaluators may view women who challenge the status quo as less competent and less fitting, reducing their chances for leadership.

### Fitting in: Maintaining the status quo through individual mobility

Rather than challenging the culture of masculine work settings, many women navigate masculine environments by assimilating into dominant norms—a pragmatic strategy with greater focus on individual mobility. One common tactic is to adopt stereotypically masculine traits to demonstrate competence and counteract negative gender‐based expectations. For instance, women leaders in masculine work settings have been found to describe themselves as more agentic than both their female junior colleagues and even their male peers (Derks et al., 2025; Ellemers et al., 2004; Faniko et al., 2021; Van Veelen & Derks, 2022). Experimental studies show that women with weaker gender identification are especially likely to emphasize their masculinity when confronted with cues of gender devaluation (Derks, Van Laar, et al., 2011).

Although women are generally more attuned to gender bias than men (Iyer & Ryan, 2009; Radke et al., 2016), women in masculine settings are often incentivized to downplay inequality and adopt “lean in” narratives oriented to individual success (Mavin & Grandy, 2018; Webber & Giuffre, 2019). In doing so, some reinforce meritocratic frames—endorsing existing arrangements, treating discrimination as isolated, doubting other women's competence/commitment or distancing from their gender identity (Cortis et al., 2021; Faniko et al., 2021; Powell et al., 2006, 2009; van Hek & Lippe, 2023). These tactics can reduce identity threat and boost perceived fit, but erode group solidarity (Becker & Tausch, 2014). This pattern—minority group members distancing from their group while supporting the status quo to pursue individual mobility—has been termed self‐group distancing (Van Veelen et al., 2020) and historically labelled the Queen Bee phenomenon (Derks et al., 2016; Mavin, 2008).

Women's efforts to fit in can be strategic in male‐dominated settings (Bryans & Mavin, 2003; Dryburg, 1999). Such environments reward traits aligned with masculine norms (Cheryan & Markus, 2020) and male gatekeepers—who hold most high‐status roles—may see women who legitimize the status quo as less threatening and more culturally compatible. Indeed, men show physiological threat responses when interacting with women who challenge (but not when they legitimize) the status quo (Domen et al., 2022). Thus, boosting a masculine leadership style, endorsing meritocracy, downplaying systemic inequality and distancing from gender‐based critiques can boost perceived competence, fit and promotability for women leaders.

From the perspective of male decision‐makers, promoting a woman who endorses existing norms can serve dual purposes. It allows men to signal support for gender diversity at the surface level—showing their commitment to equality and deflecting potential accusations of bias—while minimizing structural level change to the organizational culture. We expect that men are most likely to promote a woman who supports rather than challenges the status quo when gender inequality is highly salient and men are under higher scrutiny. For women seeking to advance in male‐dominated organizations—particularly those where gender inequality is being openly questioned—endorsing dominant norms may be more conducive to individual success than advocating for change.

## Overview of the studies and hypotheses

The present research tests whether men in masculine organizations are more likely to favour women who support—rather than challenge—the status quo. We conducted five experiments in which male (and female) participants evaluated candidates for a leadership role. Participants rated the competence and fit of male and female candidates who either challenged or supported the status quo and then made a selection decision between them. We hypothesize that women who support the status quo are evaluated more positively and are more likely to be selected for leadership than women who challenge the status quo (Hypothesis 1). In addition, we predict that while supporting the status quo leads to similarly positive evaluations for women and men, status quo‐supporting women are more likely to be selected for leadership (Hypothesis 2).

In addition, we examined whether men's preference for women who support the status quo depends on how salient and scrutinized gender inequality is. We therefore manipulated salience of gender inequality in Studies 1 and 2 and tested whether men's preference for a status quo‐supporting woman was more prevalent when gender inequality was highly salient as this may lead men to experience threats to their social identity (Hypothesis 3).

## Transparency and openness

In each reported study we describe our sampling plan and all data exclusions (if any). Materials for each study, as well as preregistrations (if applicable), can be found on https://osf.io/u8cys/overview?view_only=2403d0252dca467f8cf1c6696c62966d. All data, analysis code and research materials are available upon request from the first author. Data were analysed using SPSS version 29.0.2.0.

## STUDIES 1A AND 1B

Studies 1a and 1b tested Hypotheses 1–3. In Study 1a, male participants evaluated three candidates for a management position: two women (one supporting and one challenging the status quo) and one man (supporting the status quo). Beforehand, gender‐inequality salience was manipulated (low, medium, high). Study 1b replicated the design with female participants to examine whether effects were specific to men.

### Method

#### Participants and design

Both studies employed a 3 (candidate: SQ‐supporting woman, SQ‐challenging woman, SQ‐supporting man; within) × 3 (inequality salience: low, medium, high; between) mixed design. Participants were 90 male students in Study 1a^1^ (*M*_age_ = 21.52) and 111 female students in Study 1b (*M*_age_ = 20.60) from Leiden University, recruited for pay or course credit. Both studies were approved by the Psychology Ethics Committee at Leiden University.

Because this study was conducted before a priori sample size calculation was common practice, to assess sample size adequacy, we conducted a post‐hoc sensitivity analysis in G*Power 3.1 (Faul et al., 2007) with α = .05 and power = .80. For Study 1a (lowest N) the design was sensitive to a small‐to‐medium candidate effect (Cohen's *f* = .14, assuming *r* = .50), a medium condition effect (*f* = .27) and a medium candidate × condition interaction (*f* = .26). Thus, the study was well powered to detect candidate differences but less so for condition effects.

#### Procedure

For a full description of the stimulus materials and dependent measures, see the OSF repository.^2^ The study was presented as research on employee selection. After basic demographics, participants imagined being part of a 6‐person management team in an insurance company. Gender‐inequality salience was manipulated through team composition: low (4 men, 2 women), medium (5 men + participant; which meant all‐male for men but 5 men/1 woman for women) or high (same imbalance but explicitly criticized: “This has increasingly drawn criticism lately, as the male/female ratio in the management team does not reflect the number of women working in the rest of the organization”). To ensure engagement, participants completed three attention checks about the scenario. Participants who answered incorrectly were redirected to the vignette and asked to answer the question again. They were then asked to evaluate three candidates for an open position: two women and one man. Candidate gender was signalled by Dutch names, and interview excerpts described strengths, management style and views on gender issues. Profiles reflected (1) a SQ‐supporting woman (mainly agentic, some communal, endorsing equality as a non‐issue), (2) a similar SQ‐supporting man and (3) a SQ‐challenging woman (balanced agentic/communal, emphasizing the need for equal opportunities). Candidate order was counterbalanced. After measures, participants were debriefed.

#### Measures

All dependent measures were assessed on nine‐point scales. To test the effectiveness of the between‐subjects manipulation inequality salience, we assessed participants' *perceptions of gender inequality* (4 items, α_1a_ = .91, α_1b_ = .77; e.g., “I get the impression that a woman does not have as much chance of getting a place in the management team as a man”). After being introduced to all candidates, participants were asked to rate each candidate on *competence* (“How competent do you think [candidate name] is?”) and *team‐fit* (“To what extent do you think [candidate name] is a good fit for the management team?”). Finally, participants saw all three profiles simultaneously and were asked to *select their preferred candidate*.

### Results

#### Manipulation checks

For both male and female participants condition affected the degree to which participants perceived gender inequality (*F*_*1a*_ [2, 87] = 12.74, *p* < .001, $\eta_{p}^{2}$ = .23; *F*_*1b*_[2, 108] = 9.22, *p* < .001, $\eta_{p}^{2}$ = .15). Men and women reported perceiving less gender inequality when salience was low (*M*_*1a: men*_ = 3.79, SD = 1.62; *M*_*1b: women*_ = 3.87, SD = 1.63) compared with medium (*M*_*1a: men*_ = 5.82, SD = 1.85; *M*_*1b: women*_ = 6.26, SD = 1.53, *p's* < .001) or high (*M*_*1a: men*_ = 5.50, SD = 1.59; *M*_*1b: women*_ = 6.08, SD = 1.26, *p's* < .001). No significant differences were found between the medium and high conditions, indicating that explicitly criticizing women's underrepresentation did not increase the inequality male and female participants reported.

#### Main analyses

##### Candidate evaluations

Separate 3 (candidate) × 3 (condition) mixed ANOVAs on perceived competence and team‐fit revealed significant candidate main effects in both Studies 1a and 1b. For male participants, pairwise comparisons confirmed H1 and H2, *F*_*competence*_ (1.76,^3^ 86) = 10.88, *p* < .001, partial $\eta_{p}^{2}$ = .11; *F*_*team‐fit*_ (1.69, 87) = 13.70, *p* < .001, $\eta_{p}^{2}$ = .14. The SQ‐supporting woman was rated as more competent (*M* = 7.33, SD = 1.13) and fitting (*M* = 7.03, SD = 1.60) than the SQ‐challenging woman (*M*_*competence*_ = 6.64, SD = 1.32; *M*_*team‐fit*_ = 5.76, SD = 2.09, *p's* < .01, *p*s < .001) and equal to the SQ‐supporting man (*M*_*competence*_ = 7.20, SD = 0.98; *M*_*team‐fit*_ = 6.78, SD = 1.59, *p's* < .01) (*p*s > .58). The SQ‐challenging woman was rated as less competent and fitting than the SQ‐supporting man (*p's <* = .01).

Female participants in Study 1b however, showed a preference for the SQ‐supporting male candidate: They evaluated him as significantly more competent (*M* = 7.49, SD = 1.01) and a better fit to the team (*M* = 7.32, SD = 1.05) than both the SQ‐challenging woman (*M*_*competence*_ = 6.90, SD = 1.35; *M*_*team‐fit*_ = 6.61, SD = 1.58, *p's* < .01) and the SQ‐supporting woman (*M*_*competence*_ = 7.19, SD = 1.27; *M*_*team‐fit*_ = 6.83, SD = 1.54, *p's* < .04), *F*_*competence*_ (2107) = 11.09, *p* < .001, partial $\eta_{p}^{2}$ = .17; *F*_*team‐fit*_ (2107) = 8.95, *p* < .001, $\eta_{p}^{2}$ = .14.

In contrast to H3, no significant interactions with condition were found.

##### Candidate selection

**FIGURE 1:**
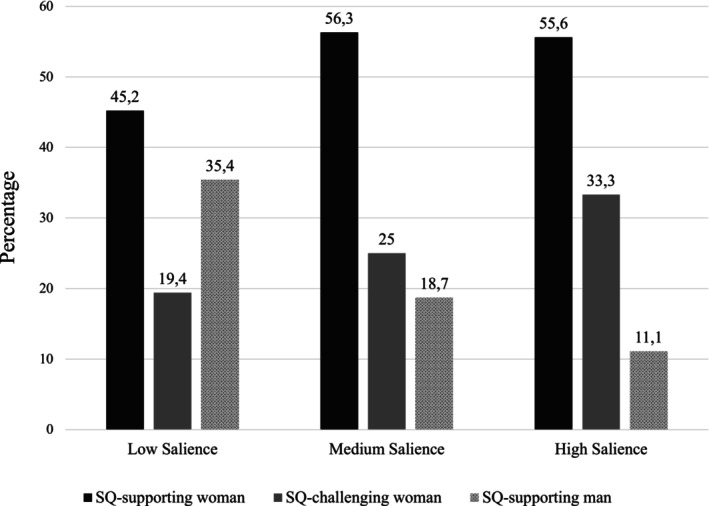
Selection rates (percentages) for each candidate across three experimental conditions (low, medium and high salience of gender inequality) in Study 1.

Among male participants in Study 1a, a chi‐square test on candidate selection revealed that the SQ‐supporting woman was the most popular choice, *χ* ^*2*^[2] = 14.6, *p* < .001. As depicted in Figure 1, more than half of participants (52%) chose her over both the SQ‐challenging woman (26%; H1) and the SQ‐supporting man (22%; H2). In contrast to H3, cross‐tabulating candidate and condition did not reveal a significant association, *χ* ^*2*^(4) = 5.69, *p* = .22.

Female participants in Study 1b, however, did not show any candidate preference, *χ* ^*2*^[2] = 1.35, *p* = .51. Each candidate was selected by about a third of participants. The inequality salience manipulation had no effect on candidate selection, *χ* ^*2*^(4) = 5.32, *p* = .26.

### Discussion

Study 1a shows that in male‐dominated contexts, men prefer women who support rather than challenge the status quo. Male raters judged a SQ‐supporting woman as more competent and a better team fit than a SQ‐challenging woman, and on par with a SQ‐supporting man. Importantly, SQ‐supporting women were much more likely to be selected than any other candidate. Study 1b found no such effect among female raters: they rated the SQ‐supporting man highest in competence and fit, and showed no selection preference. This indicates that the preference for SQ‐supporting women is specific to men.

The absence of moderation by gender‐inequality salience indicates that, contrary to H3, men did not show a stronger preference for an SQ‐legitimizing woman when women's disadvantage was made explicit. We expected heightened protection of men's status under threat, but found no support.

## STUDY 2

While Study 1 showed a clear preference for an SQ‐supporting woman, its design may have nudged men towards this choice by including two women versus one man, and two SQ‐supporting versus one SQ‐challenging candidate. To address these potential confounds, Study 2 presented four candidates varying by gender (man vs. woman) and attitude (SQ‐supporting vs. SQ‐challenging). If the SQ‐supporting woman remained preferred, this would indicate that men's choice was not simply driven by her profile being the most common combination of gender and stance.

### Method

#### Participants and design

The study had a 2 (candidate gender: woman/man; within) × 2 (candidate attitude: SQ‐supporting/SQ‐challenging; within) × 3 (inequality salience: low, medium, high; between) mixed design. One hundred students identifying as male (*M*_*age*_ = 20.87) were recruited from Leiden University in exchange for pay or to fulfil course requirements. The study was performed in accordance with all ethical requirements of the Psychology Ethics Committee at Leiden University.

Study 2 was conducted before a priori sample size calculation was common practice. Sensitivity analyses in GPower 3.1 indicated that the study (sample size of *N* = 100, alpha level = 0.05, power = 0.80) was able to detect small to medium‐sized interaction effects between candidate gender and attitude (Cohen's *f* = 0.12), and between condition and candidate (Cohen's *f* = 0.13).

### Procedure and dependent measures

The procedure was identical to Study 1, except that in Study 2 participants were presented with four different candidates for a position in the management team: In addition to the three candidates of Study 1, a fourth SQ‐challenging male candidate was added (see OSF repository for candidate profile). Dependent measures were identical to Study 1 (perceived gender inequality: α = .86). We added a manipulation check of candidate's attitude (“To what degree do you expect [candidate name] will promote the interests of women in the organization?”).

### Results

See Table 1 for descriptive statistics.

**TABLE 1:** Descriptive statistics for candidate evaluations by experimental condition (Study 2).

	Low salience	Medium salience	High salience
Competence			
SQ‐supporting woman	*M* = 7.03^a^_a_	*M* = 7.91^b^_a_	*M* = 7.03^a^_a_
	SD = 1.05	SD = 0.93	SD = 1.29
SQ‐supporting man	*M* = 7.29^a^_a_	*M* = 7.53^a^_ab_	*M* = 6.85^a^_a_
	SD = 0.94	SD = 1.27	SD = 1.21
SQ‐challenging woman	*M* = 6.62^a^_a_	*M* = 6.87^a^_b_	*M* = 7.26^a^_a_
	SD = 1.18	SD = 1.66	SD = 0.96
SQ‐challenging man	*M* = 6.65^a^_a_	*M* = 7.16^a^_b_	*M* = 7.26^a^_a_
	SD = 1.18	SD = 1.48	SD = 1.05
Fit in team			
SQ‐supporting woman	*M* = 6.65^a^_a_	*M* = 6.72^a^_ac_	*M* = 6.56^a^_a_
	SD = 1.52	SD = 1.80	SD = 1.54
SQ‐supporting man	*M* = 7.09^ab^_a_	*M* = 7.47^a^_a_	*M* = 6.50^b^_a_
	SD = 1.24	SD = 1.41	SD = 1.50
SQ‐challenging woman	*M* = 6.12^a^_a_	*M* = 5.59^a^_b_	*M* = 6.12^a^_a_
	SD = 1.82	SD = 1.97	SD = 1.92
SQ‐challenging man	*M* = 6.62^a^_a_	*M* = 6.34^a^_bc_	*M* = 6.38^a^_a_
	SD = 1.18	SD = 1.72	SD = 1.63

*Note*: Variables were measured on nine‐point scales. Different superscripts indicate significant between‐condition differences, and subscripts indicate significant within‐condition differences (*p* < .05; Bonferroni‐corrected pairwise comparisons).

#### Manipulation checks

Just like in Study 1, the manipulation of salience of inequality affected the degree to which participants perceived gender inequality, *F*(2, 97) = 26.93, *p* < .001, $\eta_{p}^{2}$ = .36. Men reported perceiving less gender inequality when salience was low (*M* = 3.87, SD = 1.63) compared with medium (*M* = 6.23, SD = 1.53, *p* < .001) or high (*M* = 6.08, SD = 1.27, *p* < .001).

The attitude manipulation successfully affected the degree to which participants expected each candidate to promote the interests of women. Participants expected SQ‐challenging candidates to do more to promote women (*M* = 7.66, SE = 0.08) than SQ‐supporting candidates (*M* = 4.96, SE = 0.15), *F*(1, 97) = 279, *p* < .001, $\eta_{p}^{2}$ = .74. However, a significant main effect of candidate gender revealed that, regardless of their attitude, participants expected female candidates to promote women more (*M* = 6.74, SE = 0.09) than male candidates would (*M* = 5.88, SE = 0.11), *F*(1, 97) = 70.77, *p* < .001, $\eta_{p}^{2}$ = .42.

#### Main analyses

##### Candidate evaluations

Evaluations were analysed with 3 (condition) × 2 (gender) × 2 (attitude) mixed ANOVAs per evaluative dimension (competence, team‐fit). Results revealed that, regardless of their gender, candidates with SQ‐supporting attitudes were seen as more competent and fitting than candidates with an SQ‐challenging attitude, *F*_*competence*_(1, 97) = 5.37, *p* = .023, $\eta_{p}^{2}$ = .05; *F*_*fit*_(1, 97) = 12.84, *p* < .001, $\eta_{p}^{2}$ = .12. Confirming H1, the SQ‐supporting woman was evaluated as more competent and fitting than the SQ‐challenging woman (*p's* < .05). Candidate gender only predicted team‐fit, with men being perceived as a better fit to the team than women, *F*(1, 97) = 11.24, *p* = .001, $\eta_{p}^{2}$ = .10. No interactions were found between candidate gender and attitude, suggesting that the effect of attitude was similar for female and male candidates.

Unlike Study 1, inequality salience moderated the effect of candidate attitude on perceived competence, *F*(2, 97) = 5.91, *p* = .004, $\eta_{p}^{2}$ = .05. SQ‐supporting candidates were seen as more competent under low and medium salience (*p*s < .02), but this advantage disappeared when inequality was explicitly criticized (*p* = .15). Thus, men's tendency to view SQ‐supporting candidates as more competent weakened under high salience of inequality.

##### Candidate selection

**FIGURE 2:**
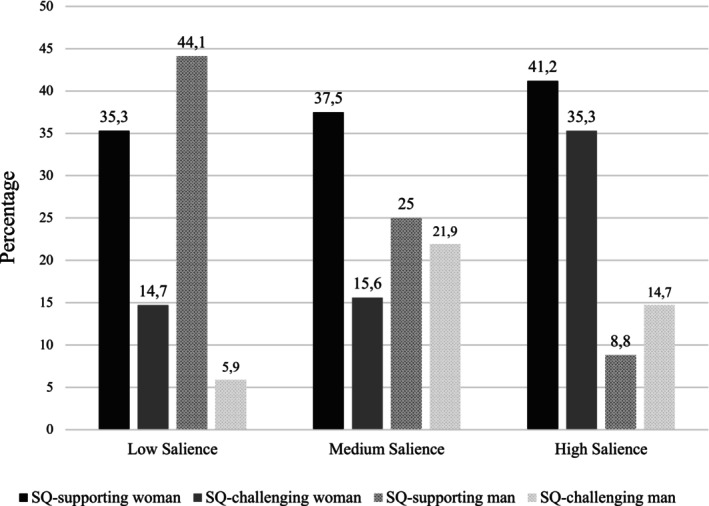
Selection rates (percentages) for each candidate across three experimental conditions (low, medium and high salience of gender inequality) in Study 2.

A Chi‐square test revealed that the SQ‐supporting woman was again the most popular choice with over a third of men (38%) selecting her over the other three candidates, *χ* ^*2*^(3) = 12, *p* = .007 (see Figure 2). Supporting H1 and H2, the SQ‐supporting woman had 2.17 times higher odds of being selected than the SQ‐challenging woman (22%) and 1.74 times higher odds than the SQ‐supporting man (26%). Cross‐tabulating candidate choice with gender‐inequality salience revealed a significant association, *χ* ^*2*^(6) = 15.49, *p* = .017. Analysis of the residuals revealed that participants in the high salience condition chose the SQ‐challenging woman significantly more often than expected (ASR = 2.3). In addition, participants in the low salience condition chose the SQ‐supporting man significantly more often than expected (ASR = 3), while participants in the high salience condition chose this candidate significantly less often than expected (ASR = −2.8). In contrast to H3, however, no effect was found for the SQ‐supporting woman who was a stable popular choice, regardless of condition.

### Discussion

Study 2 provides additional support that in male‐dominated contexts, women who support the status quo are most advantaged. Confirming H1, men rated the SQ‐supporting woman as more competent and fitting than the SQ‐challenging woman and were most likely to select her. Supporting H2, she was also more likely to be chosen than the SQ‐supporting man, despite being rated as less fitting. Study 2 ruled out the possibility that the preference for the SQ‐supporting woman in Study 1 was an artefact of the design (i.e., two women vs. one man; two SQ‐supporters vs. one challenger). By orthogonally manipulating stance and gender, we showed that it is the SQ‐supporting rather than challenging stance that drives perceptions of higher competence and fit. Nevertheless, confirming H1 the SQ‐supporting woman again emerged as the most popular candidate, indicating that her advantage reflects a genuine preference rather than a by‐product of choice architecture. A manipulation check ruled out misperceptions of her stance as participants did see the SQ‐supporting woman as less likely to promote gender equality. However, results also revealed that men expected women in general to promote gender equality more than men, consistent with prior work (Baron et al., 1991; Sterk et al., 2018).

Unlike Study 1, Study 2 showed moderating effects of inequality salience, though not as predicted. Under high salience, men were somewhat more open to SQ‐challenging candidates: they no longer rated SQ‐supporting profiles as most competent, were less likely to select the SQ‐supporting man and more likely to select the SQ‐challenging woman. Still, the SQ‐supporting woman remained preferred. Overall, this suggests that in masculine work settings, the SQ‐supporting woman functions as a compromise—she increases surface‐level diversity and deflects criticism about inequality while leaving masculine norms and hierarchies intact.

## STUDY 3

Studies 1 and 2 provided initial support for our prediction that, in masculine work settings, men would show a preference for women leaders who fit with masculine defaults and support the status quo—that is, women who will not “rock the boat”. In Studies 1 and 2 we designed the SQ‐supporting profiles based on two main aspects of self‐group distancing among women (Derks et al., 2016; Van Veelen et al., 2020), that is, emphasizing agentic self‐presentation and supporting the status quo. However, it remains unclear whether men's preference for the SQ‐supporting woman stems from her highly agentic features, her stance on gender inequality or both. Agentic masculine traits are strongly associated with perceived leadership success and are interpreted as indicators of competence, especially in masculine settings (e.g., Eagly & Karau, 2002; Heilman et al., 2024). It is therefore likely that in such settings many candidates will emphasize their masculine traits. Men's preference for an SQ‐supporting woman may reflect a genuine belief in her greater competence due to agentic qualities, rather than a desire to avoid status quo protest. Therefore, in Study 3 we examined whether, when all male and female candidates are highly agentic, women who support the status quo on gender inequality still enjoy an advantage over those who challenge it.

Second, given that inequality salience had limited effects in Studies 1 and 2, in Study 3 we kept salience of gender inequality high (i.e., participants read about an all‐male management team where the gender imbalance had been explicitly criticized). We chose to maintain this condition only as it mirrors the current debate in many male‐dominated organizations in which the underrepresentation of women in management is increasingly questioned.

Finally, an important goal of Study 3 was to provide a replication of previous findings in a well‐powered, preregistered experiment and to extend our findings beyond a student population by using a sample of adults. The study was preregistered at https://aspredicted.org/L2H_HQ1. We predicted a replication of previous findings on candidate selection, with the SQ‐supporting woman being more likely to be selected than the SQ‐challenging woman (H1) and the SQ‐supporting man (H2). Given that all candidates were presented as equally agentic, we did not expect to find differences between candidates in perceived competence. However, based on Study 2, we did predict that candidates who supported the status quo would be perceived as a better fit than candidates who challenged the status quo (H3).

### Method

#### Participants and design

We recruited 160 participants who identified as male and lived in the United States through a third‐party platform (CloudResearch) in exchange for payment. An a priori power analysis in GPower 3.1 indicated that the minimum sample size to detect a small effect (*f* = 0.1; alpha level = 0.05, power = 0.80) in a repeated‐measures design with 4 conditions was approximately 138 participants. To account for potential exclusions, we collected data from 160 participants.

Two participants who identified as non‐binary were excluded from analyses. Nineteen additional participants were excluded from analyses after responding incorrectly to attention checks (i.e., not correctly identifying there were 6 men in the management team, not selecting correct names of the candidates).^4^ The final sample consisted of 139 male participants (*M*_age_ = 37.11). The study had a 2 (candidate gender: woman/man) × 2 (candidate attitude: SQ‐supporting/SQ‐challenging) within‐subjects design. The study was approved by the Social and Behavioural Sciences Ethics Review Board at Utrecht University.

#### Procedure

The procedure closely followed Study 2 with a few exceptions (see OSF repository for stimulus materials). The original scenario was translated from Dutch to English, and gender inequality was highly salient for all participants (the team was all male and this was criticized). Participants evaluated two women and two men (as indicated by their name) who either challenged or supported the status quo through their attitudes towards gender inequality (i.e., SQ‐supporting candidates denied the existence of ongoing gender discrimination while SQ‐challenging candidates explicitly stated that gender inequality was a pressing issue). Candidate order was counterbalanced.^5^ Candidate agency was constant across candidates, with answers to questions about strengths and management style designed to convey a highly agentic profile (e.g., “I am intelligent, self‐reliant, and ambitious”). After completing dependent measures participants were thanked for their participation and fully debriefed.

#### Dependent measures

Candidate evaluations (i.e., *competence*, *team‐fit*) were measured in the same way as Studies 1 and 2. To check the effectiveness of the candidate attitude manipulation, candidates' perceived support for gender equality was measured with one item (“To what extent do you expect [candidate name] to support the implementation of policies to promote women within organizations?”). We also added a measure asking how likely they would be to *recommend* each candidate for the management team position. Finally, we asked participants to *select their preferred candidate*.^6^

### Results

Table 2 reports descriptive statistics.

**TABLE 2:** Descriptive statistics for candidate evaluations (Study 3; within subjects).

	SQ‐supporting woman	SQ‐challenging woman	SQ‐supporting man	SQ‐challenging man
Competence	*M* = 7.48^a^	*M* = 7.19^ac^	*M* = 6.97^bc^	*M* = 7.18^ac^
	SD = 1.36	SD = 1.54	SD = 1.61	SD = 1.47
Fit in team	*M* = 6.96^a^	*M* = 6.77^ac^	*M* = 6.13^bc^	*M* = 6.79^a^
	SD = 1.78	SD = 1.82	SD = 2.09	SD = 1.77
Recommend	*M* = 6.84^ac^	*M* = 6.93^a^	*M* = 5.30^b^	*M* = 6.24^c^
	SD = 12.12	SD = 2.00	SD = 2.50	SD = 1.94

*Note*: Variables were measured on nine‐point scales. Superscripts indicate significant differences between candidates (*p* < .05; pairwise comparisons, Bonferroni‐corrected).

#### Manipulation check

As expected, candidates who challenged the status quo were seen as significantly more supportive of policies to promote women within the organization (*M* = 7.94, SE = .11) than candidates who supported the status quo (*M* = 4.05, SE = .17), *F*(1, 138) = 282.54, *p* < .001, *η* ^*2*^ = .67. Like Study 2, women were seen as more supportive of these policies (*M* = 6.38, SE = .11) than men (*M* = 5.61, SE = .11), *F*(1, 138) = 35.84, *p* < .001, *η* ^2^ = .672.

#### Candidate evaluations

To examine the effects of candidate gender and attitude on competence and team‐fit, we conducted two repeated‐measures ANOVAs. Analyses yielded significant main effects of candidate gender, with women evaluated more competent and fitting than men, *F*_*competence*_(1, 138) = 8.44, *p* = .004, $\eta_{p}^{2}$ = .058, *F*_*team fit*_(1, 138) = 10.70, *p* = .001, $\eta_{p}^{2}$ = .072. These effects were qualified by an interaction between candidate gender and attitude, *F*_*competence*_(1, 138) = 10.13, *p* = .002, $\eta_{p}^{2}$ = .068, *F*_*team fit*_ (1, 138) = 19.05, *p* < .001, $\eta_{p}^{2}$ = .121. Follow up tests revealed that these interactions were driven by particularly low evaluations for the SQ‐supporting man. He was rated as less competent and a worse fit for the team than the SQ‐supporting woman (*ps* < .001), and as a worse fit than the SQ‐challenging man (*p* = .03). Women candidates were evaluated as highly competent and fitting, regardless of whether they supported or challenged the status quo. The predicted main effect of candidate attitude on perceived fit in the team (H3) was not found.

#### Candidate selection

##### Candidate recommendation

A repeated‐measures ANOVA revealed significant main effects of candidate gender, *F*(1, 138) = 65.32, *p* < .001, $\eta_{p}^{2}$ = .321, and attitude, *F*(1, 138) = 5.03, *p* = .026, $\eta_{p}^{2}$ = .035, which were qualified by a significant interaction effect, *F*(1, 138) = 13.40, *p* < .001, $\eta_{p}^{2}$ = .088. The interaction was driven by the unpopularity of the SQ‐supporting man. Participants were significantly less likely to recommend him for the management team compared with the SQ‐supporting woman (*p* < .001, supporting H2) and the SQ‐challenging man (*p* < .001). In contrast to Hypothesis 1, women received a positive recommendation regardless of whether they supported or challenged the status quo.

##### Candidate choice

**FIGURE 3:**
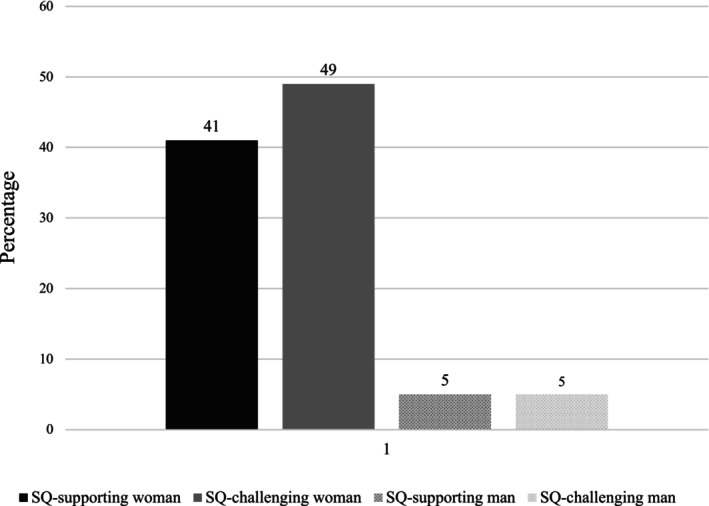
Selection rates (percentages) for each candidate in Study 3.

A Chi‐square test revealed that the four candidates were not equally popular, *χ* ^*2*^(3) = 90.38, *p* < .001 (see Figure 3). A two‐way loglinear analysis revealed that participants showed a strong preference for female over male candidates, with 89.9% of participants selecting a woman (parameter estimate = 1.06 [CI .79–1.33], *z* = 7.76, *p* < .001). No effect of candidate attitude was found (parameter estimate = 0.04, *z* = 0.32, *p =* .75). The predicted interaction between candidate gender and attitude failed to emerge (parameter estimate = 0.04, *z* = 0.32, *p =* .75). In contrast to H1, although men showed a clear preference for women candidates, they were just as like to select the woman who protected the status quo (41%) as the woman who challenged the status quo (49%).

### Discussion

A well‐powered study using a different population of participants revealed both convergent and divergent findings with Studies 1 and 2. Specifically, findings from Study 3 suggest that in a masculine context where gender inequality is highly salient, men were highly motivated to select a woman rather than a man for a leadership position. In addition, Study 3 showed an even stronger overall preference for women candidates than Studies 1 and 2, suggesting that men in this study were strongly motivated to promote women in the all‐male team. With all candidates being presented as highly agentic, whether women challenged or supported the status quo did not affect how competent and fitting they were perceived to be, nor how likely they were to be selected. This suggests that part of the unpopularity of the SQ‐challenging woman in Studies 1 and 2 was due to her less agentic self‐description rather than her stance on gender inequality.

Although supporting the status quo did not improve women's selection chances, it also did not harm them: men chose agentic women regardless of their stance. This was not because attitudes went unnoticed—participants recognized, as in Study 2, that SQ‐supporting women were less committed to gender‐equality policies—but they did not factor this into selection, reflecting ambivalence towards change. Selecting SQ‐supporting women thus enhances surface‐level diversity but leaves structural inequality intact. By contrast, men were penalized for supporting the status quo: compared with both the SQ‐supporting woman with similar views and the other male candidate, they were rated as less competent, less fitting and were rarely selected. This pattern, especially pronounced in Study 3 with adult workers, suggests greater sensitivity to men's role in maintaining inequality.

## STUDY 4

In our final study, we sought to extend our findings in several ways. First, we used a fully between‐subjects design to examine how male participants evaluate women who support (vs. challenge) the status quo when they are not comparing her to other candidates. Second, in addition to candidates who explicitly supported or challenged the status quo, we included more moderate candidates with a gender‐blind attitude whose opinion fell in between supporting and challenging the status quo.

The study was preregistered at https://aspredicted.org/RJM_SNK.^7^ Preregistered hypotheses focused specifically on the measures of candidate recommendation and selection. Although Study 3 did not provide support for H1, we preregistered our initial hypothesis predicting that SQ‐supporting women would be more likely to be recommended and selected than SQ‐challenging women. Moreover, we predicted that women who support the status quo would more likely be recommended and selected than men who support the status quo (H2). Based on the results of Study 3, we also included a new hypothesis. Specifically, we predicted that men who support the status quo would be less likely recommended and selected than men who challenge the status quo (H3). We had no a priori predictions about how the gender‐blind candidates would be viewed.

### Method

#### Participants and design

We collected data from 497 participants who completed the study through Prolific Academic in exchange for payment. An a priori power analysis in GPower 3.1 based on a small to medium effect size (*f* = 0.15) for the 2 × 3 interaction of interest indicated that the minimum sample size to obtain 80% power (alpha = .05) was approximately 435 participants. To account for potential exclusions, we increased the sample size to ~500. Recruitment was limited to adult workers who identified as male, were fluent in English and lived in the United States, United Kingdom, Ireland, Germany or the Netherlands. Two identified as women and were excluded from analyses. Following preregistered exclusion criteria,^8^ 39 participants were excluded from analyses after responding incorrectly to one or more attention/manipulation checks. Two additional participants were excluded after not consenting for their data to be used following debriefing. The final sample consisted of 447 male participants (*M*_*age*_ = 35.85). The study had a 2 (candidate gender: woman/man) × 3 (candidate attitude: SQ‐supporting/ gender‐blind /SQ‐challenging) between subjects design. The study was approved by the Social and Behavioural Sciences Ethics Review Board at Utrecht University.

#### Procedure

The procedure closely followed Study 3 with a few exceptions. The scenario was identical to Study 3, but participants were told there were three final candidates being considered for the position and that they were asked to evaluate one of these candidates (see OSF repository for stimulus materials). Each candidate profile was designed to convey a highly agentic self‐presentation. Candidate gender and attitude were manipulated in the same way as in Study 3. The newly created candidate communicated a gender‐blind ideology (Gündemir et al., 2019; e.g., “I think that organizations should be concerned with encouraging all of their employees to climb the organizational ladder. In my opinion, it is not only about gender, it is about giving people the opportunities they need to succeed.”) After completing dependent measures participants were thanked for their participation and fully debriefed.

#### Dependent measures

The attitude manipulation was checked with one question (“To what extent does this candidate support the implementation of policies to promote women within the organization”). Candidate evaluations (i.e., *competence*, *team‐fit*) were measured in the same way as Studies 1–3. *Candidate recommendation* was asked with two questions (*r* = .83, i.e., “How likely would you be to recommend the candidate for the management team position”, “How likely would you be to choose the candidate for the position”). Then participants were asked to make a dichotomous *choice* (“yes” or “no”) between selecting and not selecting the candidate they reviewed.

### Results

Table 3 reports the descriptive statistics.

**TABLE 3:** Descriptive statistics for candidate evaluations (Study 4; between subjects).

	SQ‐supporting woman	Gender‐blind woman	SQ‐challenging woman	SQ‐supporting man	Gender‐blind man	SQ‐challenging man
Competence	*M* = 7.63^a^	*M* = 7.81^a^	*M* = 7.67^a^	*M* = 7.13^c^	*M* = 7.55^ac^	*M* = 7.64^a^
	SD = 1.06	SD = 0.91	SD = 1.02	SD = 1.08	SD = 1.08	SD = 0.89
Fit in team	*M* = 7.25^ac^	*M* = 7.57^ac^	*M* = 6.99^ac^	*M* = 6.62^bc^	*M* = 7.41^a^	*M* = 7.04^ac^
	SD = 1.46	SD = 1.03	SD = 1.71	SD = 1.63	SD = 1.30	SD = 1.33
Recommend	*M* = 7.02^ac^	*M* = 7.40^a^	*M* = 6.88^ac^	*M* = 5.71^b^	*M* = 6.59^c^	*M* = 6.63^ac^
	SD = 1.53	SD = 1.09	SD = 1.75	SD = 2.05	SD = 1.43	SD = 1.41

*Note*: Variables were measured on nine‐point scales. Superscripts indicate significant differences between candidates (*p* < .05; pairwise comparisons, Bonferroni‐corrected).

#### Manipulation check

The manipulation of attitude was successful. Candidates who challenged the status quo were seen as significantly more supportive of policies to promote women within the organization (*M* = 8.40, SD = 1.09) than candidates with gender‐blind opinions (*M* = 6.18, SD = 1.5) who, in turn, were seen as more supportive than candidates who supported the status quo (*M* = 3.75, SD = 1.78), *F*(2, 441) = 370.63, *p* < .001, *η* ^*2*^ = .63. A significant interaction between attitude and gender, *F*(2, 441) = 5.19, *p* = .006, *η* ^*2*^ = .023, showed that while male and female candidates were seen as equally supportive of policies when they had an SQ‐challenging or gender‐blind attitude, when candidates had a SQ‐supporting attitude, women were seen as more likely to support policies (*M* = 4.13, SD = 1.74) than men (*M* = 3.39, SD = 1.75; *p =* .002).

#### Candidate evaluations

A two‐way ANOVA revealed that men rated female candidates as significantly more competent than male candidates, *F*(1, 441) = 7.43, *p* = .007, $\eta_{p}^{2}$ = .017. Moreover, they rated the SQ‐supporting candidates as significantly less competent than both the SQ‐challenging candidates (*p* = .01) and the gender‐blind candidates (*p* = .02), *F*(2, 441) = 4.13, *p* = .017, $\eta_{p}^{2}$ = .018. The interaction between gender and attitude was not significant, *F*(2, 441) = 2.08, *p* = .13, $\eta_{p}^{2}$ = .009. Planned pairwise comparisons revealed that whether women supported or challenged the status quo (or had a gender‐blind opinion) did not affect how competent they were perceived to be (all *p's* > .85). The SQ‐supporting man was rated as significantly less competent than the SQ‐supporting woman (*p =* .002), the SQ‐challenging man (*p* = .005) and the gender‐blind man (*p* = .03).

For team fit, the only significant effect was a main effect of candidate attitude, *F*(2, 441) = 6.35, *p* = .43, $\eta_{p}^{2}$ = .004. Men rated the gender‐blind candidates as a better fit to the team than both the SQ‐supporting (*p* = .003) and SQ‐challenging (*p* = .014) candidates. Pairwise comparisons showed that men rated the SQ‐supporting woman as a better fit to the team than the SQ‐supporting man (*p* = .03).

#### Candidate selection

##### Candidate recommendation

In addition to significant main effects of candidate gender, *F*(1, 441) = 27.70, *p* = .007, $\eta_{p}^{2}$ = .059, and attitude, *F*(2, 441) = 6.03, *p* = .003, $\eta_{p}^{2}$ = .027, analyses revealed a significant interaction between candidate gender and attitude, *F*(2, 441) = 4.31, *p* = .014, $\eta_{p}^{2}$ = .019. Similar to Study 3, this interaction was driven by the unpopularity of the SQ‐supporting man. Men were significantly less likely to recommended him for the job compared with all other candidates, including the SQ‐supporting woman (H2; *p* < .001), the SQ‐challenging man (H3; *p* < .001) and the gender‐blind man (*p =* .002). For female candidates, H1 was not supported: all women candidates were equally likely to be recommended for the job.

##### Candidate selection

**FIGURE 4:**
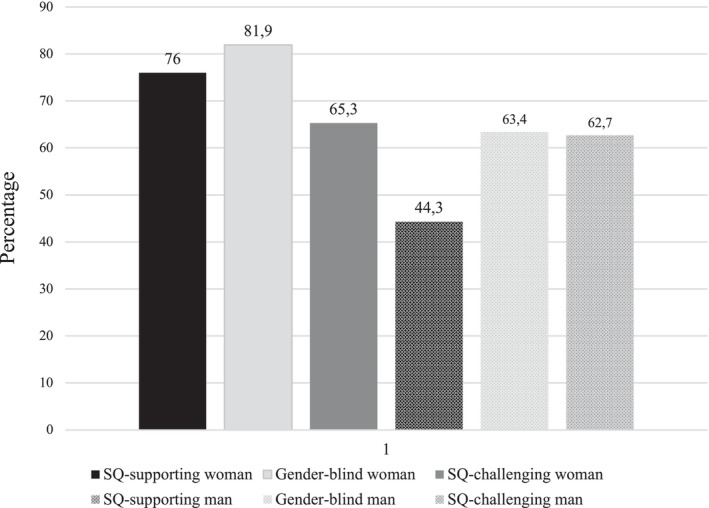
Selection rates (percentages) for each candidate in Study 4 (between subjects design).

A Chi‐square test revealed that the six candidates were not equally popular, *χ* ^*2*^(3) = 90.38, *p* < .001 (see Figure 4). To directly test our hypotheses, 2 × 2 logistic regression analyses were performed, revealing a significant interaction between gender and attitude, *Wald χ* ^*2*^(2) = 6.93, *p* = .03. No evidence was found for H1: Although the odds for being selected were 1.68 times higher for the SQ‐supporting woman than the woman who challenged the status quo, this effect was not significant, *Wald χ* ^*2*^(1) = 2.04, *p* = .15. Interestingly, men were most likely to select the gender‐blind woman (81.9%). She had 2.4 times higher odds of being selected than SQ‐challenging woman, *Wald χ* ^*2*^(1) = 5.06, *p* = .03 and similar odds to the SQ‐supporting woman, *Wald χ* ^*2*^(1) = 0.78, *p* = .38. Confirming H2, the SQ‐supporting woman had 3.98 times higher odds of being selected than the SQ‐supporting man, *Wald χ* ^*2*^(1) = 15.34, *p* < .001. Moreover, confirming H3, the odds for being selected were 2.11 times higher for the SQ‐challenging man than the SQ‐supporting man, *Wald χ* ^*2*^(1) = 5.15, *p* = .02, and 2.18 times higher for the gender‐blind man than the SQ‐supporting man, *Wald χ* ^*2*^(1) = 5.40, *p* = .02.

### Discussion

Replicating Study 3, Study 4 again shows that in a context where gender inequality is highly salient, men are more likely to recommend and select a female rather than a male candidate for a management position. In contrast to Study 3, where men selected women candidates regardless of their attitude towards the status quo, Study 4 revealed a preference among men, albeit not one that we predicted. Rather than showing a preference for a woman with a strong explicit stance on gender inequality (either downplaying or challenging) men showed a preference for a more moderate woman with a gender‐blind stance—they rated her as the best fit to the team and were more likely to select her than the woman who explicitly challenged the status quo. This finding suggests that men who were confronted with gender inequality were motivated to hire a woman rather than a man for their team (improving surface‐level gender diversity), but at the same time, selected a woman who was rather ambivalent and unlikely to actively promote change to the status quo.

Although these results were not what we predicted, they suggest that for women, the more moderate gender‐blind stance on gender inequality may be most effective. A possible explanation for this might be that this profile better fits with how women at work are prescribed to behave (e.g., nice and accommodating rather than confrontational and outspoken; Rudman et al., 2012). Indeed, the gender‐blind woman was seen as a better fit to the team than both other female candidates and had higher odds of being selected than the SQ‐challenging woman. This suggests that while communicating attitudes that clearly support the status quo (e.g., explicitly denying that gender inequality is still an issue) holds no advantage (nor disadvantage) for women in a setting where gender inequality is already on the agenda, communicating more moderate attitudes and suggesting not to emphasize gender may be most advantageous. Nevertheless, although the gender‐blind candidate's attitude was less outspoken, their gender‐blind stance can be interpreted as subtle way to maintain the gendered power structure and limit real organizational change. Further research should examine this possibility.

Replicating Study 3, men were clearly disadvantaged when they explicitly supported the status quo. Compared with the SQ‐supporting woman who communicated similar attitudes, and compared with the other male candidates, men rated him as less competent less fitting and were unlikely to select him. This again shows that while women may ‘get away’ with explicitly supporting the status quo, men do not.

## GENERAL DISCUSSION

In this research, we examined how both men and women contribute to the maintenance or disruption of gendered power dynamics in leadership, particularly within masculine organizational contexts. Across four experimental studies, we found that although men responded to calls for greater gender diversity by selecting female (rather than male) candidates for leadership roles, in three out of four experiments with men they consistently favoured women who supported the status quo over those who challenged masculine norms and advocated for change. These findings highlight how gender inequality can be subtly reinforced through the strategic decisions made by both men and women, even as organizations appear to diversify leadership.

Our work offers three key theoretical contributions. First, it demonstrates that male gatekeepers' support for gender equality is often conditional: although men were more likely to promote women over men for a leadership role, they favoured female candidates who conformed to masculine defaults and upheld existing power structures rather than those who offered more communal leadership and advocated for systemic change. Second, it reveals that women's self‐group distancing and endorsement of meritocratic narratives are not only coping strategies but also effective tactics for gaining leadership opportunities in male‐dominated settings, even when gender inequality is explicitly criticized. Third, it highlights that endorsing more “moderate”, gender‐blind attitudes—while seemingly neutral—may be a particularly effective strategy for individual women but can nevertheless undermine gender equality by subtly reinforcing meritocratic narratives that obscure structural barriers.

### Men's conditional support for gender equality

Across studies, male participants favoured female candidates who signalled support for the status quo. In Studies 1, 2 and 4, men rated women who explicitly (Studies 1 & 2: SQ‐supporting) or implicitly (Study 4: gender‐blindness) supported the status quo more positively than women who advocated for change and were more likely to select them for a leadership role. The advantage for women who supported the status quo was stronger in Studies 1 and 2—where candidates both conformed to masculine defaults (by emphasizing an agentic rather than communal leadership style) and endorsed the status quo—than in Study 4, where all candidates were equally masculine and differed only in their stance on gender inequality. For women seeking leadership roles, this suggests that presenting themselves as agentic—aligning with masculine defaults—while supporting the status quo may offer the most likely path to selection. Our findings further suggest that this preference was not due to lack of awareness: participants recognized the gender imbalance within their hypothetical teams and acknowledged that the women they selected were less likely to advance gender equality than other available candidates. This awareness emerged both when participants could directly compare candidates' attitudes (Studies 2 and 3) and when no direct comparison was possible (Study 4). Although a clear preference for a status quo‐supporting woman did not emerge in Study 3, men's indifference towards female candidates' stances on gender suggests an underlying ambivalence towards meaningful change, even in the face of visible inequality.

Why might men prefer status quo‐supporting over status quo‐challenging women leaders? Prior research suggests that men experience threat when confronted with women who challenge existing gender hierarchies but feel less threatened—and may even feel affirmed—by women who legitimize the status quo (Domen et al., 2022). Building on these findings, future research could examine whether different forms of social identity threat contribute to men's preference for women who uphold existing power structures. Such threats may stem from multiple concerns, including fear of losing group status (i.e., status threat; Dover et al., 2016; Ellemers et al., 2002), challenges to the values and norms that define masculine organizational cultures (i.e., symbolic threat; Stephan & Stephan, 2000) or concerns about group image and moral standing (i.e., image threat; Ellemers et al., 2002). In Studies 1 and 2 we manipulated the salience of gender inequality with the aim of triggering identity threat but did not find strong effects, possibly due to limited sample size and low power. Future studies could experimentally manipulate these distinct threats to men's gender identity to clarify whether—and how—different forms of threat shape men's leadership preferences, particularly the tendency to favour women who reinforce, rather than challenge, masculine norms.

Notably, while men favoured women who supported the status quo, they strongly rejected male candidates who denied ongoing gender inequality and promoted meritocratic narratives. These results suggest that men may experience discomfort when their male peers legitimize existing hierarchies. Consistent with past work on categorization threat (Ellemers et al., 2002), men may worry that when other men endorse the status quo, they themselves will be seen as complicit in maintaining inequality simply because they are male. This form of social identity threat may prompt self‐group distancing in men, as reflected in men's rejection of male candidates who reinforce masculine norms. However, the preference for status quo‐supporting women suggests that men are comfortable with the message when delivered by women but react negatively when it comes from other men.

Taken together, these findings point to a pattern of constrained or performative allyship (Kutlaca & Radke, 2023), wherein symbolic inclusion is permitted, but deeper, transformative change—and open acknowledgement of systemic inequality—is resisted.

### The strategic advantage of supporting the status quo for women leaders

Our results also offer new insights into the strategic trade‐offs women face in masculine work settings. While prior research shows that women may engage in individual mobility strategies to get ahead — such as self‐group distancing (also labelled ‘Queen Bee’ behaviour), adopting behaviours aligned with masculine norms and distancing themselves from other women to advance their careers (Derks et al., 2016; Van Veelen et al., 2020), the present research is the first to demonstrate that these strategies are indeed effective in securing leadership opportunities. In Studies 1 and 2, women who endorsed the status quo by displaying agentic leadership and downplaying ongoing gender inequality were consistently rated as more competent, perceived as a better organizational fit and were more likely to be selected than women with more communal leadership styles and who challenged existing norms. Importantly, our findings underscore the unique strategic advantage of downplaying the need for gender diversity efforts: even when agency was held constant, women who implicitly denied ongoing gender inequality (with a gender‐blind narrative) were more likely to be selected for leadership roles than women who acknowledged and sought to address it. Moreover, the results also help explain why women may distance themselves from other women and align with the status quo: doing so can be a successful strategy for career advancement in masculine organizations.

Our findings suggest that for women, adopting a gender‐blind perspective—advocating equality while downplaying gender differences (Gündemir et al., 2019)—may be more effective than explicitly supporting the status quo. In Study 4, a gender‐blind woman was seen as the best fit and most likely to be selected, compared with both SQ‐supporting and SQ‐challenging women. This moderate stance may align with prescriptive norms encouraging women to be agreeable rather than confrontational, helping them avoid backlash (Rudman et al., 2012). Yet, while gender‐blindness can reduce stereotype salience and offer short‐term benefits (Martin & Phillips, 2017), it also obscures structural barriers and undermines systemic change (Gündemir et al., 2019). In sum, although gender‐blind and SQ‐supporting strategies may advance individual women's careers, they risk reinforcing cultures that disadvantage women as a group.

### Limitations and future directions

A key limitation of the present research is the vignette‐based nature of our design. Participants were asked to imagine being part of a management team and selecting a new member without any real consequences for themselves or others. Such scenarios inevitably simplify the complexity, pressures and stakes of actual organizational decision‐making. Moreover, because participants knew the situation was hypothetical, their responses may have been influenced by normative or socially desirable considerations—particularly in Studies 1–3, where exposure to multiple candidate profiles may have made the study's aims easier to infer. While demand characteristics could partly account for the general preference for female candidates, they are less likely to explain the preference for the SQ‐supporting over the SQ‐challenging woman found in studies 1, 2 and 4. Future research should therefore examine these dynamics in real organizational contexts—for example, by analysing actual selection decisions or conducting field experiments in male‐dominated workplaces—to assess whether the patterns observed here extend beyond imagined scenarios.

A second limitation concerns our manipulation of gender‐inequality salience and sample size in Studies 1a, 1b and 2. Manipulation checks indicated little distinction between medium and high salience and even the ‘low’ condition still reflected substantial inequality (2 of 6 women). It remains unclear whether effects would differ in the absence of inequality (e.g., a 50/50 team), where men may feel less need to protect a privileged status quo—an idea tentatively supported by the greater selection of the SQ‐supporting man in the low salience condition of Study 1. In addition, the modest sample sizes in Studies 1 and 2 limited power to detect effects of salience. Although some effects emerged (e.g., greater salience increased preference for an SQ‐challenging woman and reduced preference for an SQ‐supporting man), conclusions about null effects should be drawn with caution.

While the overall pattern revealed a preference for women who supported the status quo, there was also variation among male participants. In particular, when gender inequality was made highly salient and explicitly criticized, a subset of men chose female leaders who advocated for change. These findings suggest that men's responses to gender diversity pressures are not uniform. Future research should explore individual differences that may predict men's allyship behaviours, such as gender identification strength, ingroup vs. outgroup‐focused motivations and moral beliefs (Kutlaca & Radke, 2023). Prior work suggests that men who are less strongly identified with their gender are less concerned about group status loss, hold more positive attitudes towards individuals who challenge the status quo and exhibit physiological responses associated with challenge rather than threat when confronted with a woman who opposes gender inequality (Domen et al., 2022). Similarly, categorization threat (Ellemers et al., 2002) may motivate some men to distance themselves from peers who reinforce masculine norms, aligning instead with women advocating for equality. Understanding the individual‐ and contextual‐level factors that influence whether men become transformative rather than merely performative allies represents an important avenue for future research.

Furthermore, while our findings offer insight into the strategic trade‐offs women face when presenting themselves for leadership roles, the context of evaluation likely plays a critical role. Distancing from other women and supporting dominant norms can increase women's chances of advancement, but the effectiveness of this strategy may depend on the composition of the evaluating audience. Study 1b with female participants showed that, contrary to men, women did not exhibit a preference for female candidates who supported, rather than challenged, the status quo. This suggests that women's self‐presentation strategies may be more consequential when evaluated by male‐dominated audiences. Future research should examine whether women anticipate evaluators' likely reactions to different self‐presentation tactics and whether they adjust their expressed attitudes and leadership styles accordingly.

### Practical implications

Our findings highlight the double bind women face in masculine organizations. Women are expected to promote gender equality and show communal behaviour, yet those who do so—hereby challenging the status quo—are less likely to be selected for leadership. Conversely, women who align with masculine norms and distance themselves from other women may advance but risk being labelled “queen bees” (Derks et al., 2025). This trade‐off means that gaining leadership through male approval can come at the cost of peer support, hindering women's ability to build the networks needed to succeed. Indeed, research shows that in male‐dominated settings, women derogate senior women who adopt masculine behaviours (Ely, 1994).

More broadly, our results caution that increasing women's representation in leadership is insufficient if organizations continue to favour those who reinforce existing norms. Without cultural change, such symbolic inclusion may entrench inequality. Efforts to diversify leadership must therefore be paired with initiatives that transform organizational norms, challenge meritocratic myths and address subtle biases shaping perceptions of leadership potential.

### Conclusion

The current studies reveal how gendered power dynamics are subtly sustained in masculine organizations. Extending prior work on women's strategic conformity to masculine norms, we show that men actively reward such behaviour, reinforcing existing hierarchies. Across four studies, we show how men's gatekeeping preferences and women's strategic self‐presentation interact to sustain existing power structures in male‐dominated contexts. These dynamics highlight the limitations of diversity efforts that focus solely on increasing the numerical representation of women without addressing the cultural norms that shape who is seen as leadership material. True progress towards gender equality will require not only diversifying leadership ranks but also transforming the organizational cultures in which leadership is defined and enacted.

## AUTHOR CONTRIBUTIONS

**Belle Derks:** Conceptualization; investigation; funding acquisition; writing – original draft; methodology; visualization; writing – review and editing; formal analysis; project administration; data curation; resources; supervision. **Francesca Manzi:** Conceptualization; writing – review and editing; methodology; investigation; writing – original draft; formal analysis; project administration; data curation. **Colette van Laar:** Conceptualization; funding acquisition; methodology; writing – review and editing. **Naomi Ellemers:** Conceptualization; methodology; writing – review and editing.

## Data Availability

The datasets and accompanying analysis scripts as well as the materials described in the methods sections are available upon request from the first author.

## Footnotes

^1^
One additional participant completed the study but was excluded from analyses after spending less than 7 s on the screens containing the experimental manipulation.

^2^
Studies 1 and 2 were part of a larger project on the effects of salience of gender inequality on mens personal and social identity. As such, the experiment also included additional measures that are not directly related to the focus of the current manuscript. More information is available upon request from the first author.

^3^
In all mixed ANOVAs reported in this paper, Greenhouse–Geisser corrections were applied when the sphericity assumption was violated.

^4^
Our preregistration planned to exclude participants who misidentified candidates attitudes toward gender diversity (9‐point scale, high = supportive). However, up to 43% misclassified one or more candidates, often rating SQ candidates above the midpoint, suggesting the item reflected participants interpretations rather than comprehension. We therefore deviated from preregistration and excluded participants only on the first two criteria. Responses to this item are reported as a manipulation check.

^5^
In contrast to Studies 1 and 2, where participants were first presented with all candidates before evaluating them one by one, in Study 3 participants evaluated each candidate immediately after presentation.

^6^
In addition to the outcomes reported here, Studies 3 and 4 also included exploratory measures that are not reported in this paper. More information can be obtained from the first author.

^7^
There are 4 hypotheses in the preregistration. To create fit with the other studies, we reordered preregistered hypotheses 2, 3 and 4. Because preregistered H1 was the interaction effect of candidate gender and attitude, and preregistered H2, H3 and H4 focused on planned contrasts within this interaction, we chose not the present the preregistered interaction effect as a separate hypothesis.

^8^
Although the pre‐registration mentioned 3 inclusion criteria (incorrect responses on 2 attention checks and the perceived attitude of the candidate towards gender diversity initiatives), to be consistent with Study 3 we decided to perform exclusions based on correct responses on the first two attention checks only. Candidates perceived attitude is reported in the results section.

## References

[bjso70053-bib-0001] AAUW . (2022). Barriers & bias: The status of women in leadership. AAUW: Empowering Women Since 1881. https://www.aauw.org/resources/research/barrier‐bias/

[bjso70053-bib-0002] Arvate, P. R. , Galilea, G. W. , & Todescat, I. (2018). The queen bee: A myth? The effect of top‐level female leadership on subordinate females. The Leadership Quarterly, 29(5), 533–548. 10.1016/j.leaqua.2018.03.002

[bjso70053-bib-0003] Baron, R. S. , Burgess, M. L. , & Kao, C. F. (1991). Detecting and labeling prejudice: Do female perpetrators go undetected? Personality and Social Psychology Bulletin, 17(2), 115–123. 10.1177/014616729101700201

[bjso70053-bib-0004] Becker, J. C. , & Tausch, N. (2014). When group memberships are negative: The concept, measurement, and behavioral implications of psychological disidentification. Self and Identity, 13(3), 294–321. 10.1080/15298868.2013.819991

[bjso70053-bib-0005] Becker, J. C. , Zawadzki, S. J. , & Shields, S. A. (2014). Confronting and reducing sexism: A call for research on intervention. Journal of Social Issues, 70(4), 603–614. 10.1111/josi.12081

[bjso70053-bib-0006] Benschop, Y. , Holgersson, C. , van den Brink, M. , & Wahl, A. (2015). Future challenges for practices of diversity management in organizations. In R. Bendl , I. Bleijenbergh , E. Henttonen , & A. J. Mills (Eds.), The Oxford handbook of diversity in organizations (pp. 553–574). Oxford University Press. 10.1093/oxfordhb/9780199679805.013.24

[bjso70053-bib-0007] Biernat, M. , & Kobrynowicz, D. (1997). Gender‐ and race‐based standards of competence: Lower minimum standards but higher ability standards for devalued groups. Journal of Personality and Social Psychology, 72(3), 544–557. 10.1037/0022-3514.72.3.544 9120783

[bjso70053-bib-0008] Brescoll, V. L. , & Uhlmann, E. L. (2008). Can an angry woman get ahead? Status conferral, gender, and expression of emotion in the workplace. Psychological Science, 19(3), 268–275. 10.1111/j.1467-9280.2008.02079.x 18315800

[bjso70053-bib-0010] Bryans, P. , & Mavin, S. (2003). Women learning to become managers: Learning to fit in or to play a different game? Management Learning, 34(1), 111–134. 10.1177/1350507603034001133

[bjso70053-bib-0011] Castilla, E. J. , & Benard, S. (2010). The paradox of meritocracy in organizations. Administrative Science Quarterly, 55(4), 543–576. 10.2189/asqu.2010.55.4.543

[bjso70053-bib-0012] Catalyst . (2018). Sexual harassment prevention in the workplace . https://www.catalyst.org/insights/2018/sexual‐harassment‐prevention‐workplace

[bjso70053-bib-0013] Catalyst . (2024a). Women in STEM: Science, tech, engineering, and mathematics . https://www.catalyst.org/insights/2024/women‐in‐stem

[bjso70053-bib-0014] Catalyst . (2024b). Women in the United States workforce . https://www.catalyst.org/research/women‐in‐the‐united‐states‐workforce/

[bjso70053-bib-0015] Chang, E. H. , Milkman, K. L. , Chugh, D. , & Akinola, M. (2019). Diversity thresholds: How social norms, visibility, and scrutiny relate to group composition. Academy of Management Journal, 62(1), 144–171. 10.5465/amj.2017.0440

[bjso70053-bib-0016] Cheryan, S. , & Markus, H. R. (2020). Masculine defaults: Identifying and mitigating hidden cultural biases. Psychological Review, 127(6), 1022–1052. 10.1037/rev0000209 32804526

[bjso70053-bib-0017] Cheryan, S. , Ziegler, S. A. , Montoya, A. K. , & Jiang, L. (2017). Why are some STEM fields more gender balanced than others? Psychological Bulletin, 143(1), 1–35. 10.1037/bul0000052 27732018

[bjso70053-bib-0018] Cortina, L. M. (2008). Unseen injustice: Incivility as modern discrimination in organizations. Academy of Management Review, 33(1), 55–75. 10.2307/20159376

[bjso70053-bib-0019] Cortis, N. , Foley, M. , & Williamson, S. (2021). Change agents or defending the status quo? How senior leaders frame workplace gender equality. Gender, Work and Organization, 29(1), 17–34. 10.1111/gwao.12742

[bjso70053-bib-0020] Derks, B. , Ellemers, N. , Van Laar, C. , & De Groot, K. (2011). Do sexist organizational cultures create the queen bee? British Journal of Social Psychology, 50(3), 519–535. 10.1348/014466610X525280 21884548

[bjso70053-bib-0021] Derks, B. , Manzi, F. , Van Laar, C. , & Faniko, K. (2025). Do not blame ‘queen bees’ for gender inequality in academia. Nature Human Behaviour, 9, 227. 10.1038/s41562-024-02100-6 39815010

[bjso70053-bib-0022] Derks, B. , Van Laar, C. , & Ellemers, N. (2016). The queen bee phenomenon: Why women leaders distance themselves from junior women. The Leadership Quarterly, 27(3), 456–469. 10.1016/j.leaqua.2015.12.007

[bjso70053-bib-0023] Derks, B. , Van Laar, C. , Ellemers, N. , & De Groot, K. (2011). Gender‐bias primes elicit queen‐bee responses among senior policewomen. Psychological Science, 22(10), 1243–1249. 10.1177/0956797611417258 21873568

[bjso70053-bib-0024] Dodd, E. H. , Giuliano, T. A. , Boutell, J. M. , & Moran, B. E. (2001). Respected or rejected: Perceptions of women who confront sexist remarks. Sex Roles, 45(9–10), 567–577. 10.1023/A:1014866915741

[bjso70053-bib-0025] Domen, I. , Scheepers, D. , Derks, B. , & Van Veelen, R. (2022). It's a man's world; right? How women's opinions about gender inequality affect physiological responses in men. Group Processes & Intergroup Relations, 25(3), 703–726. 10.1177/13684302211042669

[bjso70053-bib-0026] Dover, T. L. , Major, B. , & Kaiser, C. R. (2016). Members of high‐status groups are threatened by pro‐diversity organizational messages. Journal of Experimental Social Psychology, 62, 58–67. 10.1016/j.jesp.2015.10.006

[bjso70053-bib-0027] Drury, B. J. , & Kaiser, C. R. (2014). Allies against sexism: The role of men in confronting sexism. Journal of Social Issues, 70(4), 637–652. 10.1111/josi.12083

[bjso70053-bib-0028] Dryburgh, H. (1999). Work hard, play hard: Women and professionalization in engineering—Adapting to the culture. Gender & Society, 13(5), 664–682. 10.1177/089124399013005006

[bjso70053-bib-0029] Eagly, A. H. , & Carli, L. L. (2003). The female leadership advantage: An evaluation of the evidence. The Leadership Quarterly, 14(6), 807–834. 10.1016/j.leaqua.2003.09.004

[bjso70053-bib-0031] Eagly, A. H. , & Karau, S. J. (2002). Role congruity theory of prejudice toward female leaders. Psychological Review, 109(3), 573–598. 10.1037/0033-295X.109.3.573 12088246

[bjso70053-bib-0032] Eliezer, D. , & Major, B. (2012). It's not your fault: The social costs of claiming discrimination on behalf of someone else. Group Processes & Intergroup Relations, 15(4), 487–502. 10.1177/1368430211432894

[bjso70053-bib-0033] Ellemers, N. (2018). Gender stereotypes. Annual Review of Psychology, 69, 275–298. 10.1146/annurev-psych-122216-011719 28961059

[bjso70053-bib-0034] Ellemers, N. , Spears, R. , & Doosje, B. (1997). Sticking together or falling apart: In‐group identification as a psychological determinant of group commitment versus individual mobility. Journal of Personality and Social Psychology, 72(3), 617–626. 10.1037/0022-3514.72.3.617

[bjso70053-bib-0035] Ellemers, N. , Spears, R. , & Doosje, B. (2002). Self and social identity. Annual Review of Psychology, 53(1), 161–186. 10.1146/annurev.psych.53.100901.135228 11752483

[bjso70053-bib-0036] Ellemers, N. , Van Den Heuvel, H. , De Gilder, D. , Maass, A. , & Bonvini, A. (2004). The underrepresentation of women in science: Differential commitment or the queen bee syndrome? British Journal of Social Psychology, 43(3), 315–338. 10.1348/0144666042037999 15479533

[bjso70053-bib-0037] Ely, R. J. (1994). The effects of organizational demographics and social identity on relationships among professional women. Administrative Science Quarterly, 39(2), 203–238. 10.2307/2393234

[bjso70053-bib-0039] European Institute for Gender Equality . (2022). Gender balance in business and finance: December 2022. Publications Office of the European Union. 10.2839/487444

[bjso70053-bib-0040] Faniko, K. , Ellemers, N. , & Derks, B. (2021). The queen bee phenomenon in academia 15 years after: Does it still exist, and if so, why? British Journal of Social Psychology, 60(2), 383–399. 10.1111/bjso.12408 32696985 PMC8246980

[bjso70053-bib-0041] Faul, F. , Erdfelder, E. , Lang, A.‐G. , & Buchner, A. (2007). G*power 3: A flexible statistical power analysis program for the social, behavioral, and biomedical sciences. Behavior Research Methods, 39(2), 175–191. 10.3758/BF03193146 17695343

[bjso70053-bib-0042] Fritz, C. , & van Knippenberg, D. (2017). Gender and leadership aspiration: The impact of organisational identification. Leadership and Organization Development Journal, 38(8), 1018–1037. 10.1108/LODJ-05-2016-0120

[bjso70053-bib-0043] Gardner, D. M. , & Ryan, A. M. (2020). What's in it for you? Demographics and self‐interest perceptions in diversity promotion. Journal of Applied Psychology, 105(9), 1062–1072. 10.1037/apl0000478 31916784

[bjso70053-bib-0044] Gündemir, S. , Martin, A. E. , & Homan, A. C. (2019). Understanding diversity ideologies from the target's perspective: A review and future directions. Frontiers in Psychology, 10, 282. 10.3389/fpsyg.2019.00282 30873065 PMC6400841

[bjso70053-bib-0045] Heilman, M. E. , Caleo, S. , & Manzi, F. (2024). Women at work: Pathways from gender stereotypes to gender bias and discrimination. Annual Review of Organizational Psychology and Organizational Behavior, 11, 165–192. 10.1146/annurev-orgpsych-110721-034105

[bjso70053-bib-0046] Heilman, M. E. , & Manzi, F. (2022). Revisiting Schein's think manager‐think male study. In K. Steffens , R. Niklas , F. Rink , & M. Ryan (Eds.), Organizational psychology: Revisiting the classic studies. Psychology: Revisiting the Classic Studies. SAGE Publications.

[bjso70053-bib-0047] Heine, S. J. , Proulx, T. , & Vohs, K. D. (2006). The meaning maintenance model: On the coherence of social motivations. Personality and Social Psychology Review, 10(2), 88–110. 10.1207/s15327957pspr1002_1 16768649

[bjso70053-bib-0048] Hekman, D. R. , Johnson, S. K. , Der Foo, M. , & Yang, W. (2017). Does diversity‐valuing behavior result in diminished performance ratings for non‐white and female leaders? Academy of Management Journal, 60(2), 771–797. 10.5465/amj.2014.0538

[bjso70053-bib-0049] Iyer, A. (2022). Understanding advantaged groups' opposition to diversity, equity, and inclusion (DEI) policies: The role of perceived threat. Social and Personality Psychology Compass, 16(5), e12666. 10.1111/spc3.12666

[bjso70053-bib-0050] Iyer, A. , & Ryan, M. K. (2009). Why do men and women challenge gender discrimination in the workplace? The role of group status and in‐group identification in predicting pathways to collective action. Journal of Social Issues, 65(4), 791–814. 10.1111/j.1540-4560.2009.01625.x

[bjso70053-bib-0051] Joshi, A. , Oh, S. , & DesJardine, M. (2022). A new perspective on gender bias in the upper echelons: Why stakeholder variability matters. Academy of Management Review, 49(2), 322–343. 10.5465/amr.2021.0131

[bjso70053-bib-0052] Jost, J. T. , & Banaji, M. R. (1994). The role of stereotyping in system‐justification and the production of false consciousness. British Journal of Social Psychology, 33(1), 1–27. 10.1111/j.2044-8309.1994.tb01008.x

[bjso70053-bib-0053] Kanitz, R. , Reinwald, M. , Gonzalez, K. , Burmeister, A. , Song, Y. , & Hoegl, M. (2024). Supportive, resistant, or both? A person‐centric view on employee responses to diversity initiatives. Journal of Applied Psychology, 109(10), 1635–1658. 10.1037/apl0001190 38619475

[bjso70053-bib-0054] Kay, A. C. , Gaucher, D. , Peach, J. M. , Laurin, K. , Friesen, J. , Zanna, M. P. , & Spencer, S. J. (2009). Inequality, discrimination, and the power of the status quo: Direct evidence for a motivation to see the way things are as the way they should be. Journal of Personality and Social Psychology, 97(3), 421–434. 10.1037/a0015997 19685999

[bjso70053-bib-0055] Kelan, E. K. , & Wratil, P. (2018). Post‐heroic leadership, tempered radicalism and senior leaders as change agents for gender equality. European Management Review, 15(1), 5–18. 10.1111/emre.12117

[bjso70053-bib-0056] Kim, J. Y. , Fitzsimons, G. M. , & Kay, A. C. (2018). Lean in messages increase attributions of women's responsibility for gender inequality. Journal of Personality and Social Psychology, 115(6), 974–1001. 10.1037/pspa0000129 30550322

[bjso70053-bib-0057] Koenig, A. M. , & Eagly, A. H. (2014). Evidence for the social role theory of stereotype content: Observations of groups' roles shape stereotypes. Journal of Personality and Social Psychology, 107(3), 371–392. 10.1037/a0037215 25133722

[bjso70053-bib-0058] Kratz, J. (2024). History of DEI: Why it matters for the future. Forbes. https://www.forbes.com/sites/juliekratz/2024/12/29/history‐of‐dei‐why‐it‐matters‐for‐the‐future/

[bjso70053-bib-0059] Krivkovich, A. , Field, E. , Yee, L. , McConnell, M. , & Smith, H. (2024). Women in the workplace 2024: The 10th‐anniversary report. McKinsey & Company. https://www.mckinsey.com/featured‐insights/diversity‐and‐inclusion/women‐in‐the‐workplace

[bjso70053-bib-0060] Kutlaca, M. , & Radke, H. R. M. (2023). Towards an understanding of performative allyship: Definition, antecedents and consequences. Social and Personality Psychology Compass, 17(2), e12724. 10.1111/spc3.12724

[bjso70053-bib-0061] Leslie, L. M. , Kim, Y. L. , & Yeager, E. R. (2024). Diversity initiatives: Intended and unintended effects. Current Opinion in Psychology, 61, 101942. 10.1016/j.copsyc.2024.101942 39591801

[bjso70053-bib-0062] Leslie, S. , Cimpian, A. , Meyer, M. , & Freeland, E. (2015). Expectations of brilliance underlie gender distributions across academic disciplines. Science, 347(6219), 262–265. 10.1126/science.1261375 25593183

[bjso70053-bib-0063] Martin, A. E. , & Phillips, K. W. (2017). What “blindness” to gender differences helps women see and do: Implications for confidence, agency, and action in male‐dominated environments. Organizational Behavior and Human Decision Processes, 142, 28–44. 10.1016/j.obhdp.2017.07.004

[bjso70053-bib-0065] Mavin, S. (2008). Queen bees, wannabes, and afraid to bees: No more ‘best enemies’ for women in management. British Journal of Management, 19, 575–584. 10.1111/j.1467-8551.2008.00573.x

[bjso70053-bib-0066] Mavin, S. , & Grandy, G. (2018). Women leaders, self‐body‐care and corporate moderate feminism: An (im)perfect place for feminism. Gender, Work and Organization, 26(11), 1546–1561. 10.1111/gwao.12292

[bjso70053-bib-0067] McCoy, S. K. , & Major, B. (2007). Priming meritocracy and the psychological justification of inequality. Journal of Experimental Social Psychology, 43(3), 341–351. 10.1016/j.jesp.2006.04.009

[bjso70053-bib-0069] Moser, C. E. , & Branscombe, N. R. (2022). Male allies at work: Gender‐equality supportive men reduce negative underrepresentation effects among women. Social Psychological and Personality Science, 13(2), 372–381. 10.1177/19485506211033748

[bjso70053-bib-0070] Ortiz‐Ospina, E. , Tzvetkova, S. , & Roser, M. (2018). Women's employment. *Our World in Data* . https://ourworldindata.org/female‐labor‐supply

[bjso70053-bib-0071] Penner, A. M. , Petersen, T. , Hermansen, A. S. , Rainey, A. , Boza, I. , Elvira, M. M. , Godechot, O. , Hällsten, M. , Henriksen, L. F. , Hou, F. , Mrčela, A. K. , King, J. , Kodama, N. , Kristal, T. , Křížková, A. , Lippényi, Z. , Melzer, S. M. , Mun, E. , Apascaritei, P. , … Tufail, Z. (2023). Within‐job gender pay inequality in 15 countries. Nature Human Behaviour, 7, 184–189. 10.1038/s41562-022-01470-z PMC9957724 36424396

[bjso70053-bib-0072] Pietri, E. S. , Moser, C. E. , Derricks, V. , & Johnson, I. R. (2024). A framework for understanding effective allyship. Nature Reviews Psychology, 3(10), 686–700. 10.1038/s44159-024-00359-0

[bjso70053-bib-0073] Post, C. (2015). When is female leadership an advantage? Coordination requirements, team cohesion, and team interaction norms. Journal of Organizational Behavior, 36, 1153–1175. 10.1002/job.2031

[bjso70053-bib-0075] Powell, A. , Bagilhole, B. M. , & Dainty, A. R. J. (2006). The problem of women's assimilation into UK engineering cultures: Can critical mass work? Equal Opportunities International, 25(8), 688–699. 10.1108/02610150610719146

[bjso70053-bib-0074] Powell, A. , Bagilhole, B. , & Dainty, A. (2009). How women engineers do and undo gender: Consequences for gender equality. Gender, Work and Organization, 16(4), 411–428. 10.1111/j.1468-0432.2008.00406.x

[bjso70053-bib-0076] Radke, H. R. M. , Hornsey, M. J. , & Barlow, F. K. (2016). Barriers to women engaging in collective action to overcome sexism. American Psychologist, 71(9), 863–874. 10.1037/a0040345 28032778

[bjso70053-bib-0077] Rudman, L. A. , & Glick, P. (1999). Feminized management and backlash toward agentic women: The hidden costs to women of a kinder, gentler image of middle managers. Journal of Personality and Social Psychology, 77(5), 1004–1010. 10.1037/0022-3514.77.5.1004 10573877

[bjso70053-bib-0078] Rudman, L. A. , & Glick, P. (2001). Prescriptive gender stereotypes and backlash toward agentic women. Journal of Social Issues, 57(4), 743–762. 10.1111/0022-4537.00239

[bjso70053-bib-0079] Rudman, L. A. , Moss‐Racusin, C. A. , Phelan, J. E. , & Nauts, S. (2012). Status incongruity and backlash effects: Defending the gender hierarchy motivates prejudice against female leaders. Journal of Experimental Social Psychology, 48(1), 165–179. 10.1016/j.jesp.2011.10.008

[bjso70053-bib-0080] Ryan, M. K. , & Morgenroth, T. (2024). Why we should stop trying to fix women: How context shapes and constrains women's career trajectories. Annual Review of Psychology, 75(1), 555–572. 10.1146/annurev-psych-032620-030938 38236650

[bjso70053-bib-0081] Sargent, A. C. , Shanock, L. G. , Banks, G. C. , & Yavorsky, J. E. (2022). How gender matters: A conceptual and process model for family‐supportive supervisor behaviors. Human Resource Management Review, 32(4), 100880. 10.1016/j.hrmr.2021.100880

[bjso70053-bib-0082] Sealy, R. , & Singh, V. (2010). The importance of role models and demographic context for senior women's work identity development. International Journal of Management Reviews, 12(3), 284–300. 10.1111/j.1468-2370.2009.00262.x

[bjso70053-bib-0100] Sherf, E. N. , Tangirala, S. , & Weber, K. C. (2017). It is not my place! Psychological standing and men's voice and participation in gender‐parity initiatives. Organization Science, 28(2), 193–210. 10.1287/orsc.2017.1118

[bjso70053-bib-0083] Shuman, E. , Knowles, E. , & Goldenberg, A. (2023). To overcome resistance to DEI, understand what's driving it. Harvard Business Review. https://hbr.org/2023/03/to‐overcome‐resistance‐to‐dei‐understand‐whats‐driving‐it

[bjso70053-bib-0084] Stainback, K. , & Kwon, S. (2012). Female leaders, organizational power, and sex segregation. The Annals of the American Academy of Political and Social Science, 639(1), 217–235. 10.1177/0002716211421868

[bjso70053-bib-0085] Stephan, W. G. , & Stephan, C. W. (2000). An integrated threat theory of prejudice. In S. Oskamp (Ed.), Reducing prejudice and discrimination (pp. 23–45). Lawrence Erlbaum Associates Publishers.

[bjso70053-bib-0086] Sterk, N. , Meeussen, L. , & Van Laar, C. (2018). Perpetuating inequality: Junior women do not see queen bee behavior as negative but are nonetheless negatively affected by it. Frontiers in Psychology, 9, 1690. 10.3389/fpsyg.2018.01690 30294289 PMC6159757

[bjso70053-bib-0087] UN Women . (2024). Facts and figures: Women's leadership and political participation. UN Women. https://www.unwomen.org/en/what‐we‐do/leadership‐and‐political‐participation/facts‐and‐figures

[bjso70053-bib-0088] UN Women . (2025). Facts and figures: Women's leadership and political participation. UN Women. https://www.unwomen.org/en/articles/facts‐and‐figures/facts‐and‐figures‐womens‐leadership‐and‐political‐participation

[bjso70053-bib-0089] van der Hek, M. , & Lippe, T. (2023). Why female employees do not earn more under a female manager: A mixed‐method study. Work, Employment and Society, 37(6), 1462–1479. 10.1177/09500170221083971

[bjso70053-bib-0090] Van Veelen, R. , & Derks, B. (2022). Academics as agentic superheroes: Female academics' lack of fit with the agentic stereotype of success limits their career advancement. British Journal of Social Psychology, 61(3), 748–767. 10.1111/bjso.12515 34935167

[bjso70053-bib-0091] Van Veelen, R. , Veldman, J. , Van Laar, C. , & Derks, B. (2020). Distancing from a stigmatized social identity: State of the art and future research agenda on self‐group distancing. European Journal of Social Psychology, 50(6), 1089–1107. 10.1002/ejsp.2714

[bjso70053-bib-0092] Veldman, M. , Doolaard, S. , Bosker, R. , & Snijders, T. (2020). Young children working together. Cooperative learning effects on group work of children in grade 1 of primary education. Learning and Instruction, 67, 101308. 10.1016/j.learninstruc.2020.101308

[bjso70053-bib-0093] Vial, A. C. , & Cowgill, C. M. (2022). Heavier lies her crown: Gendered patterns of leader emotional labor and their downstream effects. Frontiers in Psychology, 13, 849566. 10.3389/fpsyg.2022.849566 36106035 PMC9465331

[bjso70053-bib-0095] Wallen, J. (2002). Balancing work and family: The role of the workplace. Allyn & Bacon.

[bjso70053-bib-0096] Webber, G. R. , & Giuffre, P. (2019). Women's relationships with women at work: Barriers to solidarity. Sociology Compass, 13(6), e12698. 10.1111/soc4.12698

[bjso70053-bib-0098] Zehnter, M. K. , Manzi, F. , Shrout, P. E. , & Heilman, M. E. (2021). Belief in sexism shift: Defining a new form of contemporary sexism and introducing the belief in sexism shift scale (BSS scale). PLoS One, 16(3), e0248374. 10.1371/journal.pone.0248374 33705476 PMC7951888

[bjso70053-bib-0099] Zimmermann, F. (2022). Managing the gender wage gap—How female managers influence the gender wage gap among workers. European Sociological Review, 38(3), 355–370. 10.1093/esr/jcab046

